# phyloPipeR: An R Package for End-to-End Phylogenetic Reconstruction and Tree Comparison

**DOI:** 10.3390/cimb48060600

**Published:** 2026-06-05

**Authors:** Feifei Li, Yue Zou, Tong Li, Lingling Xie, Dandan Liu, Kunhong Song, Yanting Luo, Dan Qin, Youjin Hao, Bo Li

**Affiliations:** 1 Computational and Integrative Biology Group, College of Life Sciences, Chongqing Normal University, Chongqing 401331, China; 2023051301066@stu.cqnu.edu.cn (F.L.); 2025110513077@stu.cqnu.edu.cn (Y.Z.); 2024210513012@stu.cqnu.edu.cn (T.L.); 2024110513055@stu.cqnu.edu.cn (L.X.); 2023051301033@stu.cqnu.edu.cn (D.L.); 2024210513025@stu.cqnu.edu.cn (K.S.); 2025210513018@stu.cqnu.edu.cn (Y.L.); nkhyjin@cqnu.edu.cn (Y.H.); 2 Department of Biochemical and Cellular Pharmacology, Genentech Inc., One DNA Way, South San Francisco, CA 94080, USA; qin.dan@gene.com; 3 Chongqing Higher Education Engineering Research Center for Digital Intelligence in Education, Chongqing Normal University, Chongqing 401331, China

**Keywords:** phylogenetic reconstruction, phylogenomics, R package, workflow integration, tree comparison, coalescent analysis

## Abstract

Phylogenetic reconstruction is a multi-step process that typically involves sequence retrieval, alignment, trimming, and tree inference, often requiring the integration of multiple independent tools. This fragmented workflow increases technical complexity and limits reproducibility, particularly in large-scale analyses. Here, we present phyloPipeR, an R package that provides an integrated and automated framework for end-to-end phylogenetic analysis and tree comparison within a unified environment. The phyloPipeR enables complete workflows from ortholog retrieval to tree inference and quantitative comparison, while also supporting modular execution of individual steps. The package implements multiple phylogenetic inference methods and supports both concatenation and coalescent strategies for multi-gene analyses. By integrating tree reconstruction and quantitative comparison within a single framework, phyloPipeR improves reproducibility, reduces technical barriers, and provides a scalable solution for systematic and integrative evolutionary studies.

## 1. Introduction

Phylogenetic reconstruction, a fundamental approach for studying evolutionary relationships among species and genes, has been widely used in evolutionary biology and comparative genomics [1]. As the volume of genomic and transcriptomic data continues to expand, the scope and scale of phylogenetic analyses have grown substantially [2,3,4,5], placing increasing demands on computational methods and analytical workflows. Over the past decades, a diverse set of phylogenetic inference methods has been developed, including distance-based methods such as Neighbor-Joining (NJ) [6], character-based methods like Maximum Parsimony (MP) [7], likelihood-based approaches such as Maximum Likelihood (ML) [8], Bayesian inference (BI) methods [9], and clustering-based approaches like UPGMA [10]. Together, these methods constitute the core methodological framework for modern phylogenetic analysis.

In practice, conducting a complete phylogenetic analysis remains a multi-step process that relies on the integration of several independent tools [11,12]. A typical workflow includes sequence retrieval, multiple sequence alignment, alignment trimming, and phylogenetic tree inference, each often implemented in different software environments. These tools vary in input formats, parameter settings, and execution environments, requiring users to manually coordinate data transfer and format conversion across stages. As a result, the analytical process becomes technically demanding and error-prone, particularly when handling multiple genes or large datasets.

The increasing scale and complexity of phylogenomic datasets present additional challenges to existing analytical workflows. In large-scale analyses, coordinating multiple independent tools reduces efficiency and makes it difficult to maintain consistent analytical settings across different stages. Differences in software implementation and data processing can introduce variability, complicating the reproducibility of results even when identical parameters are applied [13]. Moreover, key analytical components—such as ortholog identification, alignment, model testing, and tree inference—are often distributed across separate platforms [14], further increasing the burden of workflow management and limiting scalability in systematic studies.

Limitations associated with fragmented workflows also extend to the R environment, a platform widely used for data analysis in evolutionary biology. Several R packages provide important functionality for phylogenetic analysis, including tree manipulation (ape, v5.8.1) [15], phylogenetic inference (phangorn, v2.12.1) [16], visualization and annotation of phylogenetic trees (ggtree, v3.12.0) [17]. While these tools are well established and widely adopted, they are primarily designed to address specific analytical tasks rather than to support an integrated workflow. As a result, building a fully automated and reproducible phylogenetic pipeline still requires manual coordination of multiple tools, particularly in large-scale gene-level analyses and systematic tree comparisons [12].

Beyond tree reconstruction, quantitative comparison of phylogenetic trees has become increasingly important. Differences between gene trees and species trees may arise from processes such as incomplete lineage sorting [18], gene duplication and loss [19], horizontal gene transfer [20], or variation in evolutionary constraints [21]. Additionally, phylogenies inferred from nucleotide and protein sequences may exhibit distinct topological patterns [22]. Systematic evaluation of these differences requires quantitative measures of tree similarity. However, these comparison approaches are rarely integrated into existing analytical workflows [12], particularly within the R ecosystem, limiting their application to large-scale and reproducible analyses.

To address these challenges, we developed phyloPipeR, an R package that provides an integrated and automated workflow for phylogenetic reconstruction and tree comparison. This package enables end-to-end analyses, from ortholog retrieval and sequence processing to tree inference and quantitative tree comparison, within a unified R environment. By consolidating these steps into a single framework, phyloPipeR reduces technical complexity, improves reproducibility, and supports large-scale evolutionary analyses. Notably, phyloPipeR includes a dedicated module for quantitative tree comparison, enabling systematic evaluation of phylogenetic concordance across genes and datasets.

## 2. Materials and Methods

### 2.1. Conceptual Framework and System Design of phyloPipeR

**Figure 1:**
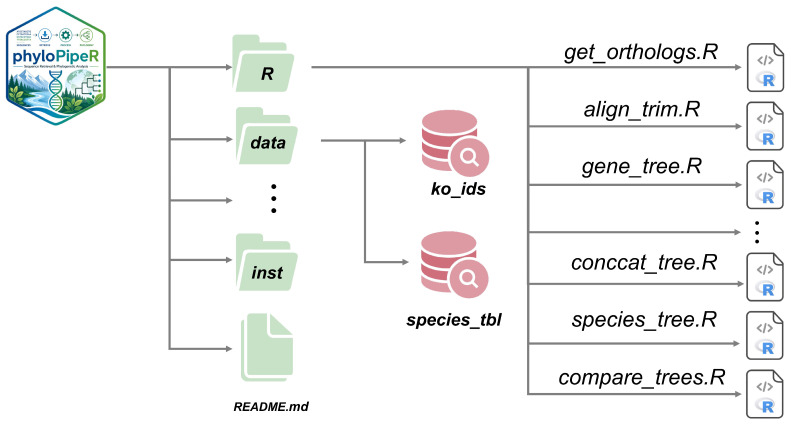
The complete architecture of the phyloPipeR package. The diagram illustrates the organization of source code, internal datasets, and supporting files according to standard R package conventions, including the *R*/, *data*/, and *inst*/ directories.

The complete architecture of phyloPipeR is illustrated in Figure 1. The package is organized according to standard R package conventions, with a clear separation of functional components, data resources, and supporting files. This structured design provides a stable foundation for integrating diverse phylogenetic analysis tasks within a unified computational environment.

At the system level, phyloPipeR adopts a modular architecture in which core analytical functionalities are encapsulated into independent yet interoperable components. The *R/* directory contains the main implementation of analytical functions, each corresponding to a specific stage of the phylogenetic workflow, while the *data/* directory stores curated internal datasets that support sequence retrieval and downstream analysis. Additional resources, including reproducibility scripts and auxiliary files, are organized within the *inst/* directory, ensuring transparency and ease of reuse. This organization enables a clear separation between data management, algorithm implementation, and workflow control, reducing interdependencies among components and improving maintainability. At the same time, standardized data structures are used across modules to facilitate seamless data exchange between different analytical steps, thereby minimizing the need for manual data handling.

### 2.2. Computational Workflow for End-to-End Phylogenetic Analysis

**Figure 2:**
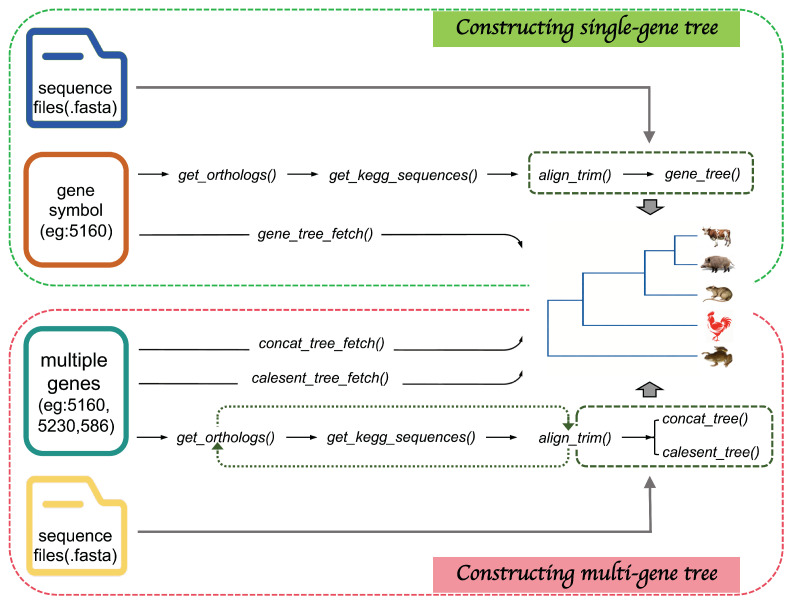
Computational workflow of phylogenetic tree in phyloPipeR. This module represents the tree construction component of the package and supports two analytical branches for phylogenetic reconstruction. The single-gene workflow performs direct tree inference following ortholog retrieval, whereas the multi-gene workflow includes sequence alignment and trimming, followed by tree reconstruction using concatenation-based or coalescent-based approaches.

The computational framework implemented in phyloPipeR consists of two major components: phylogenetic tree construction and tree comparison. The tree construction workflow is illustrated in Figure 2, while tree comparison is implemented as a complementary analytical module.

In phyloPipeR, the tree-construction workflow is organized into two analytical branches, supporting phylogenetic inference based on either single-gene or multi-gene datasets. For single-gene analysis, the workflow begins with user-specified gene identifiers and target species, followed by retrieval of orthologous sequences from public databases. Phylogenetic trees are then directly reconstructed using integrated inference procedures, enabling rapid generation of gene-level phylogenies with minimal preprocessing. For multi-gene analysis, phyloPipeR adopts a more comprehensive workflow based on established phylogenomic strategies [23]. Orthologous sequences from multiple genes are first collected and subjected to multiple sequence alignment and automated trimming to ensure data quality. Based on the processed sequence sets, phylogenetic trees can be reconstructed using either concatenation-based approaches [24], which combine multiple loci into a single supermatrix, or coalescent-based approaches [25], which explicitly account for gene tree heterogeneity. This design provides flexible strategies for modeling evolutionary relationships across loci under different evolutionary assumptions. Beyond public database retrieval, phyloPipeR supports user-provided sequence datasets, including local FASTA files and R sequence objects, and users can resume analyses from saved intermediate outputs generated by individual modules.

Additionally, phyloPipeR provides a dedicated module for quantitative comparison of phylogenetic trees. This module operates independently of the construction workflow and can be applied to trees generated within phyloPipeR or imported from external sources. By computing similarity metrics across trees derived from different genes, datasets, or inference methods, phyloPipeR enables systematic evaluation of phylogenetic concordance and discordance.

To further position phyloPipeR within the landscape of commonly used phylogenetic tools, we prepared an interactive comparison table named “PhyloCompare” (https://www.ciblab.net/pub/phyloCompare.html, accessed on 17 May 2026), hosted on our laboratory website. PhyloCompare summarizes key features of MEGA [26], PHYLIP [27], FastTree [28], and IQ-TREE 3 [29], including development environment, interface type, multi-gene strategy support, tree comparison metrics, visualization, reporting, and workflow integration. This comparison highlights the R-native, modular, reproducible, and teaching-friendly design of phyloPipeR.

### 2.3. Modular Implementation of Analytical Components

The analytical functionalities of phyloPipeR are organized into a set of modular components, each corresponding to a specific stage of the phylogenetic analysis workflow. These modules collectively support both phylogenetic tree construction and tree comparison, enabling flexible yet coherent analytical processes within a unified framework. The main functions associated with each module are summarized in Table 1.

At the initial stage, phyloPipeR provides modules for ortholog identification and sequence retrieval, allowing users to obtain homologous sequences across multiple species from public databases. These modules serve as the entry point of the workflow, transforming user-defined gene and species inputs into standardized sequence datasets for downstream analysis. To improve input data quality, phyloPipeR includes a duplicate-checking step that detects duplicated sequence identifiers or identical sequence records and returns a warning before tree inference.

Following sequence acquisition, preprocessing modules perform multiple sequence alignment and trimming. For alignment, phyloPipeR provides three commonly used global alignment algorithms, including ClustalW, ClustalOmega, and MUSCLE. Users can adjust key parameters, such as *gapOpening*, *gapExtension*, and *maxiters*, to accommodate datasets with different sequence divergence levels or length variation. For trimming, phyloPipeR applies coverage-based trimming rather than removing all gap-containing columns. Users can control the maximum allowed gap proportion at sequence ends (*gap.end*) and internal regions (*gap.mid*), thereby removing poorly aligned regions while retaining informative alignment columns. These steps are designed to improve data quality by removing poorly aligned or ambiguous regions, thereby ensuring that subsequent phylogenetic inference is based on reliable sequence information.

For phylogenetic reconstruction, phyloPipeR implements both single-gene and multi-gene analysis modules. The single-gene module enables direct inference of gene trees, while the multi-gene module supports two complementary strategies: concatenation-based approaches, which combine multiple loci into a single supermatrix, and coalescent-based approaches, which account for gene tree heterogeneity. For concatenation-based analysis, phyloPipeR does not assume that every orthologous gene is available in all selected species. If an ortholog is absent in one or more species, those species are automatically excluded from the corresponding tree reconstruction step, and a warning message is returned to inform the user. Tree reconstruction in phyloPipeR is implemented within the R environment mainly through established packages such as ape and phangorn, rather than by calling external software such as IQ-TREE 3. The package supports NJ, ML, MP, UPGMA, and BI methods through a unified interface. To improve model specification, users can set the model parameter to select appropriate substitution models for DNA or protein sequences, such as JC, K80, HKY, TN93, GTR, JTT, WAG, or LG, depending on the data type and supported inference method.

In addition to tree reconstruction, phyloPipeR provides a dedicated module for quantitative phylogenetic tree comparison. This module provides multiple metrics for evaluating topological similarity and structural differences between trees, facilitating systematic assessment of phylogenetic concordance across genes, datasets, and inference methods.

### 2.4. Mathematical Formulation of Tree Similarity Metrics

To quantify the topological similarity and structural differences between phylogenetic trees, pholyPipeR implements three complementary metrics: Robinson–Foulds (RF) similarity [30], edge-based Jaccard similarity [31,32] and entanglement [33]. RF similarity measures differences in bipartition structures between two trees, whereas Jaccard similarity evaluates the proportion of shared internal edges or split-defining structures using an intersection-over-union formulation. Entanglement, derived from tanglegram-based comparison, provides an additional measure of structural discordance by assessing the degree of mismatch between two tree layouts. Together, these metrics provide complementary perspectives on tree similarity and enable systematic comparison of phylogenetic trees generated from different genes, datasets, or inference methods.

Here, all metrics are computed for pairs of trees defined on the same set of taxa.

#### 2.4.1. Robinson–Foulds (RF) Similarity

Robinson–Foulds (RF) distance measures the topological difference between two phylogenetic trees based on their bipartition structures. For two trees $T_{i}$ and $T_{j}$, let $S(T_{i})$ and $S(T_{j})$ denote the sets of splits induced by their internal edges [30]. For unrooted trees, each split corresponds to an internal edge, whereas for rooted trees, splits correspond to clade-defining bipartitions. Therefore, RF similarity provides a straightforward and widely accepted measure of overall topological dissimilarity, making it a suitable baseline for comparing tree structures.

Robinson–Foulds distance is defined as

(1) $$d_{RF}\left(T_{i},T_{j}\right)=|S(T_{i})\;\Delta \;S(T_{j})|$$

where Δ denotes the symmetric difference between the two split sets.

To facilitate comparison across trees, the RF similarity is defined as a normalized measure:

(2) $${Sim}_{RF}(T_{i},T_{j})=1-\frac{d_{RF}(T_{i},T_{j})}{|S(T_{i})|+|S(T_{j})|}$$

This formulation rescales the RF distance to the interval $\left[0,\;1\right]$, where a value of 1 indicates identical tree topologies.

#### 2.4.2. Edge-Based Jaccard Similarity

Edge-based Jaccard similarity quantifies the proportion of shared internal edges, splits, or clade-defining structures between two trees. Following the general Jaccard index for set similarity and its use in split-based phylogenetic tree comparison, two trees are represented as sets of internal edges or induced splits, and their similarity is calculated as the size of the intersection divided by the size of the union. Let $E(T_{i})$ and $E(T_{j})$ denote the sets of internal edges in trees $T_{i}$ and $T_{j}$, respectively. Thus, Jaccard similarity provides a normalized measure of clade overlap, allowing meaningful comparison even when trees differ in size or taxon composition.

Edge-based Jaccard similarity [31] between the two trees is defined as

(3) $$J(T_{i},T_{j})=\frac{|E(T_{i})\cap E(T_{j})|}{|E(T_{i})\cup E(T_{j})|}$$

where the numerator represents the number of shared edges and the denominator represents the total number of unique edges across the two trees. The resulting value ranges from 0 to 1, with higher values indicating greater topological similarity.

#### 2.4.3. Entanglement

While RF and Jaccard similarities capture topological agreement, they do not account for differences in tree layout. To quantify structural discordance under a fixed leaf ordering, phyloPipeR employs the entanglement metric based on tanglegram representations. Entanglement provides a complementary perspective that is sensitive to branch order and leaf interleaving, capturing topological discordance that RF and Jaccard may overlook [33].

Let $p_{i}(k)$ and $p_{j}(k)$ denote the vertical positions of leaf k in tree $T_{j}$ and $T_{j}$, respectively. The entanglement between $T_{i}$ and $T_{j}$ is defined as the normalized L-norm of the positional differences between corresponding leaves:

(4) $$E_{L}(T_{i},T_{j})=\frac{{\left(\sum_{k=1}^{n}|p_{i}(k)-p_{j}(k){|}^{L}\right)}^{1/L}}{\underset{\pi }{max}{\left(\sum_{k=1}^{n}|p_{i}(k)-p_{\pi }(k){|}^{L}\right)}^{1/L}}$$

where L≥1 controls the sensitivity to large deviations, and the denominator represents the maximum possible value L-norm over all permutations π. The entanglement value ranges from 0 to 1, with lower values indicating greater concordance between trees.

In this study, the three metrics implemented in phyloPipeR were selected to provide complementary perspectives on tree similarity. RF similarity captures differences in bipartition structures, Jaccard similarity evaluates the proportion of shared internal edges or split-defining structures using an intersection-over-union formulation, and entanglement provides a tanglegram-based measure of structural discordance between tree layouts. Other metrics, such as weighted RF distance, quartet distance, and Kendall–Colijn distance, may offer additional information by considering branch lengths, quartet relationships, or tree-shape features. These metrics are not included in the current version, but they represent useful directions for future expansion of the tree comparison module.

### 2.5. Reproducible Workflow Examples

To demonstrate the practical application of phyloPipeR, we provide representative examples covering key stages of the phylogenetic analysis workflow, including sequence retrieval, phylogenetic reconstruction, and tree comparison.

The three-letter species codes used in these examples are standardized species abbreviations recognized by the sequence retrieval module. To facilitate reproducibility, phyloPipeR provides a species annotation dataset, *species_tbl.rda*, in the package data/ directory. This dataset records the mapping among species scientific names, three-letter species abbreviations, and corresponding identifiers, and can be directly called and inspected in R using *species_tbl*.

#### 2.5.1. Sequence Retrieval

**Figure 3:**
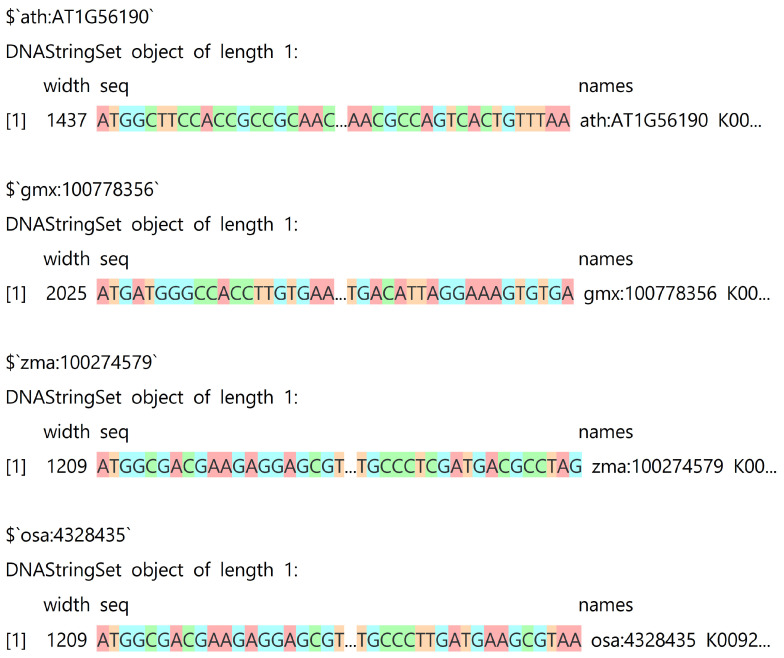
Representative R output generated from the sequence retrieval example in Listing 1. The retrieved sequences are stored as a list consisting of multiple DNAStringSet objects. Because the sequence identifiers are relatively long, the default R print method abbreviates the names field in the console display; however, the complete names are retained in the returned object. Users can inspect the combined species-code and Entrez-ID (or Locus_tag) names using *names(seq_set)*, or query the full name of a specific sequence, for example, using *names(seq_set$osa:4328435)*. This figure is intended to demonstrate successful sequence retrieval and object display in R rather than to show all metadata fields in full.

The phyloPipeR enables automated retrieval of homologous sequences from public databases based on user-defined gene identifiers and species (Listing 1). Retrieved sequences are provided as standardized objects for direct use in downstream alignment and phylogenetic analysis. Corresponding results for Listing 1 are shown in Figure 3 as visualized in the R environment.
**Listing 1.** R code for automatically sequence retrieval and output in the R environment.*# Fetching sequences* seq_set <- get_kegg_sequences(gene_ids = “K00927”, *# Input gene ID*               *# Specify the ID format of input genes*                id.type = “ko_id”,                *# Specify the type of sequences to retrieve*                seq.type = “DNA”,               *# Select species for tree construction*                species.list = c(“ath”, “gmx”, “zma”, “osa”),                *# Specify the naming format of input species*                species.type = “abbspname”) *# Output the seq_set object* print(seq_set)

#### 2.5.2. Phylogenetic Reconstruction Using Multiple Methods

**Figure 4:**
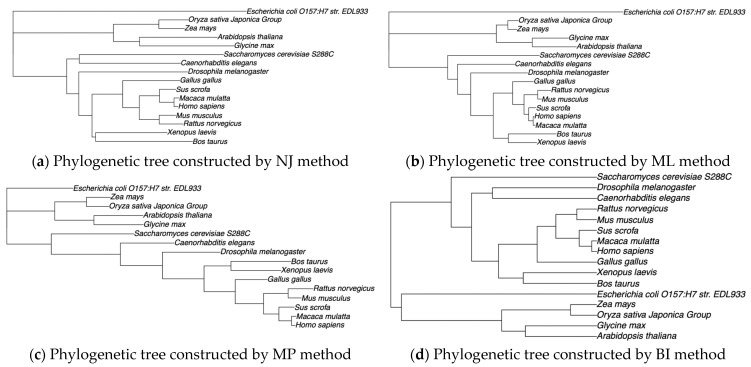
Phylogenetic trees reconstructed using different methods: (**a**) NJ; (**b**) ML; (**c**) MP; (**d**) BI. The figure is intended to illustrate the ability of phyloPipeR to generate phylogenetic trees using multiple inference methods and to provide a qualitative comparison of their overall topological patterns, rather than to quantitatively compare branch lengths across methods.

The phyloPipeR supports phylogenetic inference using multiple widely used methods within a unified interface (as shown in Listing 2). These functions generate phylogenetic trees based on different inference strategies, enabling comparative evaluation of tree topologies. Four trees built by NJ, MP, ML, and BI are shown in Figure 4.
**Listing 2.** R code for constructing phylogenetic tree construction using four major algorithms (NJ, MP, ML and BI).*# Obtain DNA sequence information* DNA_seq <- system.file(“extdata”, “DNA_seq.fas”, package = “phyloPipeR”) *# Construct phylogenetic tree using NJ method* tree1 <- gene_tree(seq.file = DNA_seq,           seq.type = “DNA”,           tree_method = “NJ”) *# Construct phylogenetic tree using ML method* tree2 <- gene_tree(seq.file = DNA_seq,           seq.type = “DNA”,           tree_method = “ML”) *# Construct phylogenetic tree using MP method* tree3 <- gene_tree(seq.file = DNA_seq,           seq.type = “DNA”,           tree_method = “MP”) *# Construct phylogenetic tree using BI method* tree4 <- gene_tree(seq.file = DNA_seq,           seq.type = “DNA”,           tree_method = “BI”)

#### 2.5.3. Quantitative Comparison of Phylogenetic Trees

**Figure 5:**
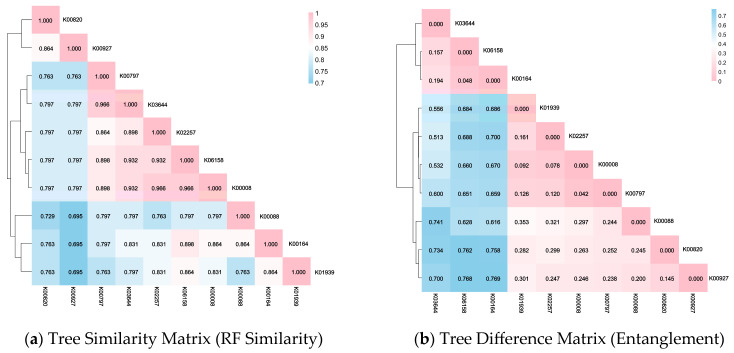
Pairwise similarity matrices comparing phylogenetic trees: (**a**) RF similarity matrix: each entry represents the Robinson–Foulds similarity between two trees, ranging from 0 (completely different topology) to 1 (identical topology). (**b**) Entanglement matrix: each entry quantifies the entanglement between two trees based on tanglegram overlap, where lower values indicate more similar branching structures.

The phyloPipeR further provides functions for systematic comparison of phylogenetic trees derived from different genes or inference methods (Listing 3). This step quantifies topological similarity and structural differences between trees, facilitating downstream comparative analyses. Figure 5 shows the tree similarity matrices derived from the comparison between two phylogenetic trees: (a) RF similarity matrix and (b) entanglement matrix.
**Listing 3.** R code for comparing two phylogenetic trees.*# List of genes for tree construction* gene_ids <- c(“K00820”, “K00088”, “K00927”, “K06158”, “K00008”,         “K00164”, “K00797”, “K01939”, “K02257”, “K03644”)  
*# Obtain DNA sequences and construct trees separately*
for (i in
1:10) {  file.path <- paste0(“sequences/”, gene_ids[i], “.fas”)  tmp <- assign(paste0(“seq”, i),          system.file(“extdata”,              file.path,               package = “phyloPipeR”))  assign(paste0(“tree”, i), gene_tree(seq.file = tmp,                   seq.type = “DNA”,                   tree_method = “UPGMA”)) }  
*# Consolidate ten phylogenetic trees* trees <- list(tree1, tree2, tree3, tree4, tree5,        tree6, tree7, tree8, tree9, tree10)  
*# Build correlation matrix for RF similarity* rf_matrix <- matrix(NA, 10, 10, dimnames = list(gene_ids, gene_ids))  
*# Build correlation matrix for entanglement* entanglement_matrix <- matrix(   NA, 10, 10, dimnames = list(gene_ids, gene_ids))  
*# Calculate correlation matrices for RF similarity and entanglement*
for (i in
1:10) {  for (j in
i:10) {   *# Comparison between different trees*   tmp <- compare_trees(trees[[i]], trees[[j]])   *# Extract RF similarity and entanglement values*   rf_value <- ifelse(is.numeric(tmp$RF_similarity), tmp$RF_similarity, NA)   entanglement_value <- ifelse(is.numeric(tmp$entanglement_value),                   tmp$entanglement_value, NA)   rf_matrix[c(i,j), c(j,i)] <- rf_value   entanglement_matrix[c(i,j), c(j,i)] <- entanglement_value  } }  
*# Assign values to diagonals of RF similarity and entanglement matrices*
diag(rf_matrix) <- 1
diag(entanglement_matrix) <- 0  
*# Plot correlation matrix heatmap (using RF similarity as an example))*
pheatmap(rf_matrix,cluster_rows = TRUE,        cluster_cols = TRUE,        display_numbers = TRUE,        number_format = “%.3f”,        number_color = “black”,        main = “Tree Similarity Matrix (RF Similarity)”,        color = colorRampPalette(c(“skyblue”, “white”, “pink”))(100),        border_color = NA,        fontsize_number = 8)

### 2.6. Implementation and Availability

The phyloPipeR package is implemented in R and is publicly available at GitHub (https://github.com/libcell/phyloPipeR, accessed on 17 May 2026). It provides an integrated workflow for molecular phylogenetics, including ortholog identification, sequence retrieval, alignment and trimming, phylogenetic inference, and quantitative tree comparison.

Installation can be performed directly from the repository using the remotes package in the R environment:

*if (!require(“remotes”)) install.packages(“remotes”)*

*remotes::install_github(“libcell/phyloPipeR”, dependencies = TRUE)*

As an alternative, phyloPipeR can also be installed using the install_ciblab utility in the R environment:

*source(“https://ciblab.net/pub/install_ciblab.R”,* *accessed on 17 May 2026)*

*install_ciblab(“phyloPipeR”)*

To facilitate reproducible deployment across different computational environments, we also provide a Docker image of phyloPipeR on Docker Hub (https://hub.docker.com/r/cqnulibcell/phylopiper, accessed on 17 May 2026). Users can download and launch the container using the following Bash commands in the terminal:

*docker pull cqnulibcell/phylopiper*

*docker run -it cqnulibcell/phylopiper:latest /bin/bash*

After installation, phyloPipeR supports both fully automated, end-to-end analyses and modular execution of individual analytical steps. Detailed documentation and reproducible workflows are available in the repository.

To further document the computational cost of phyloPipeR, we benchmarked 11 major functions implemented in the package. Each function was evaluated in three independent runs, and runtime, peak memory usage, and total allocated memory were recorded as minimum, maximum, mean, and standard deviation. The complete benchmark results, task settings, and computational environment, including R version, operating system, processor, and RAM, are provided in Supplementary Tables S1–S3.

## 3. Application Case and Results

To demonstrate the practical utility of phyloPipeR, we present an application case based on 178 universal single-copy orthologs across 16 model species. This case illustrates how phyloPipeR can be used to reconstruct DNA-based gene trees and protein-based trees, quantify tree-structure similarity among gene, protein, and species trees, cluster genes according to phylogenetic concordance patterns, and further connect these outputs with downstream functional interpretation. The enrichment and functional analyses presented below are therefore intended to demonstrate the interpretability of phyloPipeR-derived results rather than to serve as an independent biological discovery study.

### 3.1. Gene-Specific Phylogenetic Concordance Analysis

Comparative analysis of phylogenetic trees reconstructed from 178 universal single-copy orthologs across 16 model species revealed substantial heterogeneity in phylogenetic concordance. Specifically, gene trees derived from nucleotide sequences and those inferred from protein sequences exhibited varying degrees of agreement with each other and with the reference species tree.

**Figure 6:**
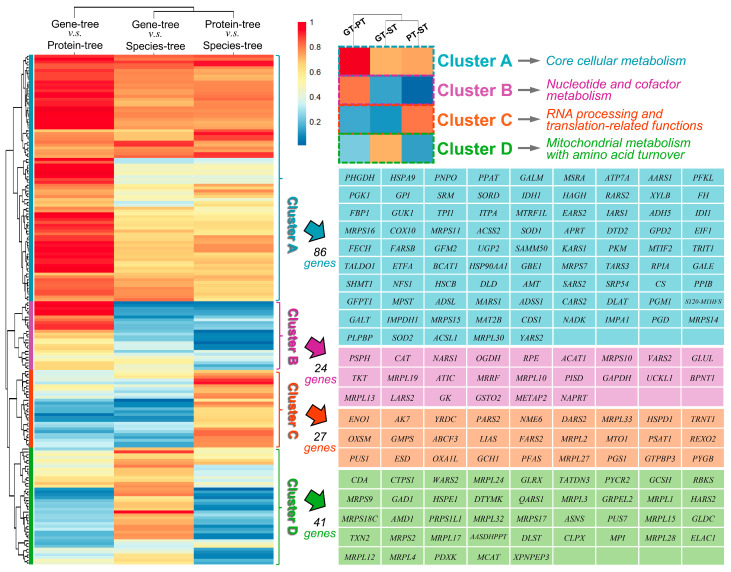
Comparative analysis of DNA-based gene trees, protein-based trees, and the reference species tree based on 178 universal single-copy orthologs from 16 model species. GT, PT, and ST denote gene tree, protein tree, and species tree, respectively. For each ortholog, GT, PT, and ST were compared pairwise, including GT vs. PT, GT vs. ST, and PT vs. ST. Tree-structure similarity was quantified as (1—entanglement), with higher values indicating greater similarity. The resulting similarity matrix was visualized using the pheatmap R package, and genes were grouped into four clusters by setting *kmeans_k* = 4. Representative functional annotations of the four clusters are summarized on the right.

**Figure 7:**
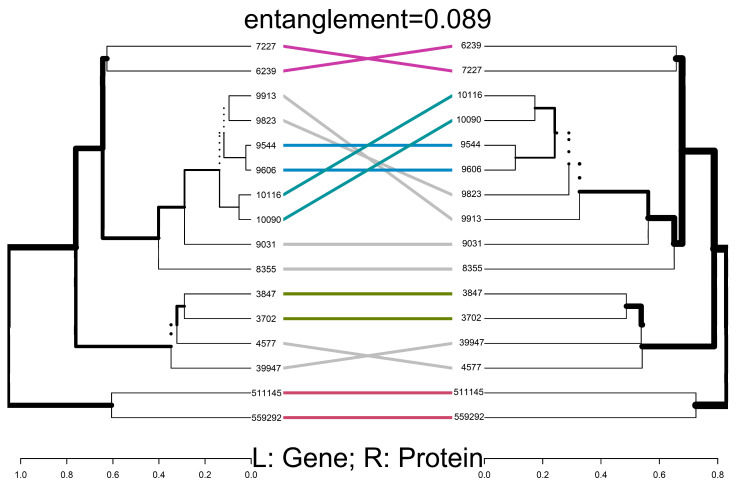
Entanglement-based comparison between the DNA-based gene tree and the protein-based tree reconstructed from the K00058 ortholog. Taxon labels are shown as Taxa IDs to provide standardized and unambiguous species identifiers. The corresponding mapping among Taxa IDs, three-letter species abbreviations, and scientific names is provided in the built-in *species_tbl.rda* dataset of the phyloPipeR package.

At the level of individual genes, phylogenetic trees inferred from DNA and protein sequences often displayed broadly similar topological structures, as illustrated by representative examples (Figure 6). However, quantitative comparisons revealed that the extent of concordance between gene trees, protein trees, and the species tree varied markedly across genes. This variability is further highlighted by entanglement analysis. For example, a comparison between the gene tree and protein tree derived from the K00058 ortholog demonstrates measurable structural differences (Figure 7), indicating that even for conserved genes, phylogenetic signals can diverge depending on the molecular data type.

These findings demonstrate that phylogenetic signals are not uniformly preserved across genes or data types, but instead reflect heterogeneous evolutionary histories across the genome.

### 3.2. Concordance-Based Gene Clustering

Clustering based on phylogenetic concordance patterns revealed four distinct groups of genes, each characterized by a specific pattern of agreement among gene trees, protein trees, and the species tree (Figure 6).

Genes in Cluster A exhibit strong concordance across all three tree types, indicating highly consistent phylogenetic signals at both nucleotide and protein levels. In contrast, Cluster B is characterized by high agreement between DNA- and protein-based trees but clear divergence from the species tree, suggesting shared gene-level signals that do not fully reflect species relationships.

Clusters C and D display reduced concordance between DNA and protein trees, but differ in their alignment with the species tree. In Cluster C, protein-based trees more closely recapitulate species-level topology, whereas in Cluster D, DNA-based trees show greater agreement with the species tree.

The above analysis of concordance patterns identifies four gene groups with distinct phylogenetic behaviors, indicating systematic variation in the representation of evolutionary signals across genes and molecular data types.

### 3.3. Functional Enrichment of phyloPipeR-Derived Gene Clusters

To illustrate the downstream interpretability of phyloPipeR outputs, we performed Gene Ontology (GO) biological process enrichment analysis on the four gene clusters derived from concordance-based clustering using the clusterProfiler [34]. This analysis was used as a downstream interpretation step rather than as a separate biological discovery workflow.

**Figure 8:**
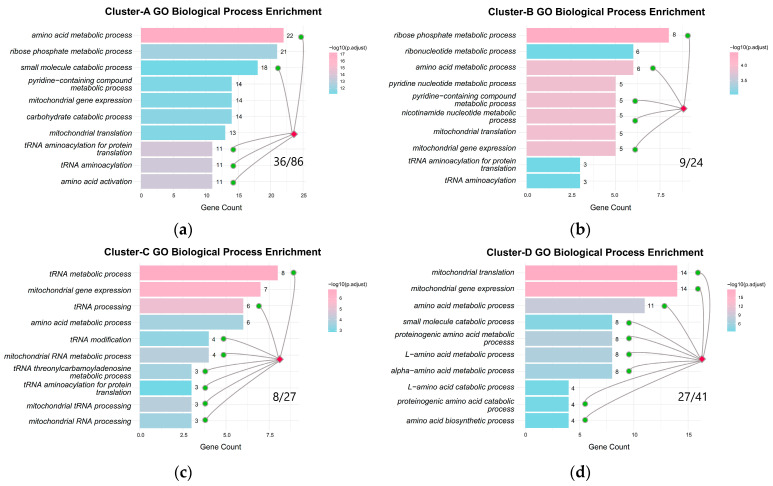
GO biological process enrichment across four gene clusters. (**a**–**d**) Enriched GO terms for Cluster-A, Cluster-B, Cluster-C, and Cluster-D, respectively. Bar length represents the number of genes annotated to each GO biological process term, with values labeled next to the bars. Green dots indicate the enriched GO terms used to summarize the representative functional categories of each cluster. Because one gene may be annotated to multiple GO terms, the gene counts in individual bars may overlap and are not expected to sum directly to the fraction shown in each panel. The fraction in each panel indicates the number of genes associated with the green-dot-highlighted enriched GO biological process terms relative to the total number of genes in the corresponding cluster: 36/86 for Cluster-A, 9/24 for Cluster-B, 8/27 for Cluster-C, and 27/41 for Cluster-D. For each bar, the color indicates enrichment significance (−log10 adjusted *p*-value).

As shown in Figure 8, the results reveal clear functional differentiation among the four clusters (Cluster-A, B, C, and D). Cluster-A was mainly enriched in core cellular metabolic processes, consistent with its high concordance among DNA-based gene trees, protein-based trees, and the reference species tree. Cluster-B was enriched in nucleotide- and cofactor-related processes, suggesting internally consistent gene-level signals that may differ from species-level topology. Cluster-C was associated with RNA processing and translation-related functions, whereas Cluster-D was enriched in mitochondrial metabolism and amino acid turnover.

Overall, these results show that phyloPipeR-derived concordance patterns can be linked to functional annotations, providing a practical route from quantitative tree comparison to biologically interpretable hypotheses.

### 3.4. Exploratory Interpretation of Concordance Patterns for Gene Clusters

As the final step of this application case, we explored whether the functional profiles of phyloPipeR-derived gene clusters could provide a biological context for the observed concordance patterns. These interpretations are intended as exploratory hypotheses rather than definitive causal conclusions.

Cluster-A, enriched in core cellular metabolic processes, showed relatively consistent signals across nucleotide-based, protein-based, and species-level trees, possibly reflecting strong and stable functional constraints. Cluster-B, associated with nucleotide and cofactor metabolism, may reflect shared functional dependencies within interconnected metabolic networks, leading to internally consistent gene-level signals that partly deviate from the species tree. Cluster-C and Cluster-D showed reduced concordance between DNA- and protein-based trees. Cluster-C, enriched in RNA processing and translation, may retain stronger phylogenetic signals at the protein level, whereas Cluster-D, associated with mitochondrial metabolism and amino acid turnover, may show relatively stronger signal retention at the nucleotide level.

Together, this application case illustrates how phyloPipeR-derived tree comparison and clustering outputs can be linked with downstream functional annotation to generate biologically interpretable hypotheses.

## 4. Discussion

In this study, we present phyloPipeR, an integrated R package that enables end-to-end phylogenetic reconstruction and quantitative tree comparison within a unified analytical framework. By consolidating sequence retrieval, alignment, tree inference, and tree comparison into a single workflow, phyloPipeR addresses persistent challenges in phylogenetic analysis, including workflow fragmentation, technical complexity, and limited support for systematic evaluation of tree concordance.

Application of phyloPipeR to 178 universal single-copy orthologs across 16 model species reveals substantial heterogeneity in phylogenetic concordance at both gene and representation levels. Rather than exhibiting uniform agreement, gene trees and protein trees varied in their consistency with the species tree, highlighting the complexity of phylogenetic signal representation across the genome. These observations emphasize that phylogenetic inference is influenced not only by methodological choices but also by gene-specific evolutionary characteristics. Functional enrichment analysis further demonstrated that differences in phylogenetic concordance are closely associated with gene function. Genes involved in core cellular processes tend to exhibit stable and consistent phylogenetic signals, whereas genes associated with metabolically interconnected pathways or specialized biological functions show greater divergence. These findings suggest that functional constraints and evolutionary regimes play a critical role in shaping the reliability of phylogenetic inference across genes.

From a methodological perspective, these findings in this study underscore the importance of integrating multiple analytical strategies when interpreting phylogenetic relationships. The phyloPipeR package enables single-gene and multi-gene phylogenetic analyses, with the latter implemented via concatenation-based and coalescent-based approaches, providing a flexible framework for exploring different evolutionary scenarios. In addition, the incorporation of quantitative tree comparison metrics enables systematic evaluation of phylogenetic concordance, which is often overlooked in conventional workflows.

Despite these advantages, several limitations should be considered. First, the current implementation relies on sequence data retrieved from public databases, which may vary in quality and annotation consistency. Second, while phyloPipeR integrates commonly used phylogenetic methods, it does not yet include advanced model selection. Third, the interpretation of tree comparison metrics may depend on the underlying tree structure and data characteristics, requiring careful consideration in specific applications. Future developments of phyloPipeR will focus on extending its methodological scope and improving usability. Potential directions include the integration of additional phylogenetic inference methods, enhanced support for large-scale datasets, and the incorporation of more advanced statistical frameworks for tree comparison. Furthermore, expanding compatibility with other bioinformatics tools and workflows will further strengthen its utility in diverse research contexts.

Collectively, phyloPipeR provides a unified and extensible platform for phylogenetic analysis, enabling both the construction and systematic evaluation of phylogenetic trees. By bridging methodological gaps and facilitating reproducible analyses, phyloPipeR has the potential to support a wide range of evolutionary and comparative genomic studies.

## 5. Conclusions

The phyloPipeR establishes a unified framework for phylogenetic analysis in R by integrating tree construction and quantitative tree comparison into a single workflow, thereby consolidating analytical steps that are typically distributed across multiple tools into a coherent and consistent analytical process. By supporting multiple inference strategies alongside complementary multi-gene reconstruction approaches, phyloPipeR enables direct comparison of alternative phylogenetic hypotheses within a shared analytical framework, while the incorporation of tree comparison metrics facilitates systematic assessment of concordance across genes, datasets, and methods. These capabilities extend conventional phylogenetic workflows by improving the comparability and interpretability of evolutionary analyses, providing a practical foundation for large-scale and integrative studies in evolutionary biology and comparative genomics.

## Figures and Tables

**Table 1:** Overview of the main functions implemented in phyloPipeR.

Function	Description
*get_orthologs*	Identifies orthologs across multiple species
*get_kegg_sequences*	Retrieving DNA/protein sequences from KEGG for selected species
*align_trim*	Conducts multiple sequence alignment followed by automated trimming
*gene_tree*	Infers phylogenetic relationships from single-gene sequence datasets
*concat_tree*	Generates concatenated phylogenies by integrating multiple gene alignments
*coalescent_tree*	Reconstructs phylogenies under a coalescent framework from multiple loci
*species_tree*	Derives species-level phylogenetic relationships from input taxa
*compare_tree*	Quantifies topological similarity and structural differences between phylogenetic trees
*gene_tree_fetch*	Performs integrated single-gene phylogenetic analysis from sequence retrieval to tree inference
*concat_tree_fetch*	Executes concatenation-based phylogenetic reconstruction across multiple genes and species
*coalescent_tree_fetch*	Executes coalescent-based phylogenetic reconstruction integrating multiple genes and species

## Data Availability

All datasets used in this article are freely available at https://github.com/libcell/phyloPipeR (accessed on 24 April 2026).

## Supplementary Materials

The following supporting information can be downloaded at: https://www.mdpi.com/article/10.3390/cimb48060600/s1.

## Abbreviations

The following abbreviations are used in this manuscript:

NJ	Neighbor-Joining
ML	Maximum Likelihood
MP	Maximum Parsimony
BI	Bayesian Inference
UPGMA	Unweighted Pair Group Method using arithmetic Averages
GT	Gene Tree
PT	Protein Tree
ST	Species Tree
